# Stress is dominant in patients with depression and chronic low back pain. A qualitative study of psychotherapeutic interventions for patients with non-specific low back pain of 3–12 months’ duration

**DOI:** 10.1186/1471-2474-13-166

**Published:** 2012-09-06

**Authors:** Hanne Ellegaard, Birthe D Pedersen

**Affiliations:** 1 Research Department, Spine Centre of Southern Denmark, Hospital Lillebaelt, Institute of Regional Health Services Research University of Southern Denmark, Østre Hougvej, 55, 5500, Middelfart, Denmark; 2 Research Unit of Nursing, Institute of Clinical Research, Faculty of Health Sciences, University of Southern Denmark, Campusvej, 55, 5230, Odense M, Denmark

**Keywords:** Psychotherapy, Chronic low back pain, Chronic pain, Depression, Stress, Qualitative method, Gestalt therapy, Somatic Experiencing® method

## Abstract

**Background:**

There is continuing uncertainty in back pain research as to which treatment is best suited to patients with non-specific chronic low back pain (CLBP). In this study, Gestalt therapy and the shock trauma method Somatic Experiencing® (SE) were used as interventions in parallel with the usual cross-disciplinary approach. The aim was to investigate how these treatments influence a patient’s capacity to cope with CLBP when it is coupled with depression.

**Methods:**

In this qualitative explorative study, a phenomenological–hermeneutic framework was adopted. Patients were recruited on the basis of following criteria: A moderate depression score of 23–30 according to the Beck Depression Inventory Scale and a pain score of 7–10 (Box scale from 0–10) and attendance at five- six psychotherapeutic sessions. Six patients participated in the study. The data was comprised of written field notes from each session, which were subsequently analysed and interpreted at three levels: naive reading, structural analysis and critical interpretation and discussion.

**Results:**

Three areas of focus emerged: the significance of previous experiences, restrictions in everyday life and restoration of inner resources during the therapy period. The study revealed a diversity of psychological stressors that related to loss and sorrow, being let down, violations, traumatic events and reduced functioning, which led to displays of distress, powerlessness, reduced self-worth, anxiety and discomfort.

Overall, the sum of the stressors together with pain and depression were shown to trigger stress symptoms. Stress was down-played in the psychotherapeutic treatment and inner resources were re-established, which manifested as increased relaxation, presence, self-worth, sense of responsibility and happiness. This, in turn, assisted the patients to better manage their CLBP.

**Conclusions:**

CLBP is a stress factor in itself but when coupled with depression, they can be regarded as two symptom complexes that mutually affect each other in negative ways. When pain, stress and depression become overwhelming and there are few internal resources available, *stress* seems to become prominent. In this study, Gestalt therapy and the SE-method may have helped to lower the six patients’ level of stress and restore their own internal resources, thereby increasing their capacity to cope with their CLBP.

## Background

This article reports on the way in which two psychotherapeutic treatments can influence a patient´s capacity to cope with non-specific chronic low back pain when it is complicated by depression. Interest in this area stems from the first author´s experience as a nurse, Gestalt therapist and Somatic Experiencing® Practitioner, working with patients who have low back pain.

Over the decades, treatment for patients with back pain has become more and more intensive; nevertheless, approximately 20% continue not to benefit from evidence-based somatic treatment [1]. With longer-term or chronic low back pain (CLBP), psychological factors often play a role, but in cross-disciplinary treatment, individual psychological intervention is rarely included. CLBP is defined as lasting more than three months.

A literature review found that epidemiological research [2] has shown a link between low back pain (LBP) and psychological factors. Depression and anxiety are thought to be the most common psychological conditions associated with LBP. The three components of LBP are described as the somatic, the depressive and the social aspects. In relation to LBP, depression is often described as being atypical as it takes the form of a so-called masked depression, often following a traumatic event. Individual psychological intervention is recommended as the primary treatment, with medical treatment secondary. Different theories [3] have been developed to explain the connection between CLBP and depression, but none is comprehensive.

A study of Gestalt therapy and chronic pain [4] reported that the most significant issue for the patient was to be understood. Individual psychotherapy was recommended with a view to understanding the patient’s subjective experiences, since everyone experiences illness differently. The study showed, furthermore, that responses depended on the severity of the illness, family background and history, networks, social class and economic situation. Furthermore, the best results were seen in those who were willing to work with their own psychological process.

In a meta-analysis of psychological interventions [5] for CLBP, the authors describe the positive effects of psychological interventions in contrast to various control groups, in the domains of pain intensity, pain-related interference, health-related quality of life, and depression. Multidisciplinary approaches that included a psychological component, when compared with active control conditions, were also noted to have positive short-term effects on pain interference and positive long-term effects on return to work.

Another meta-analysis, which focussed on patients in need of psychotherapy [6] reported that Gestalt therapy is an effective psychotherapeutic form of therapy and one that is not inferior to other comparable methods within psychotherapy.

Some people get chronic pain and depression. Chronic pain [7] is often characterized by severe pain associated with little or no discernible injury or pathology. And chronic pain is often associated with chronic stress. Evidence has been showed [8] that negative influences such as neglect and incest in childhood can lead to more violent reactions to stress later in life, with depression and anxiety more likely to develop. Furthermore, as is well known, stress coupled with depression and pain does not create a good starting-point for coping.

### Research problem and aim

The rationale for studying psychotherapeutic treatment is that there continues to be a degree of uncertainty in back pain research as to which treatment best benefits patients with a chronic and complex pain problem. It is assumed that by studying psychotherapeutic treatment, one can gain insight into, and glean new knowledge about, which conditions are significant when helping patients with CLBP and depression manage their situation better.

The aim of the study was to investigate how the selected psychotherapeutic treatments can influence a patient’s capacity to cope with CLBP when it is coupled with depression.

### Theoretical framework

Gestalt therapy [9] was chosen as the theoretical framework for the study, and this form of psychological therapy was employed as the intervention by the first author in the sessions. Gestalt means “a meaningful whole” and the whole is always more than, and different from, the sum of its parts. In Gestalt therapy, the meta-theory is existentialism, the psychological theory is Gestalt psychology and the method is phenomenological. In addition, there are diverse therapeutic techniques. The foundational idea of Gestalt therapy is that adults can choose how to lead their lives and they are at all times responsible for their own choices and actions. The phenomenological approach is a psychological method of holistically describing experienced phenomena. Increased acknowledgement of oneself often occurs in here-and-now situations, where client/patient and therapist meet.

The Somatic Experiencing® method (SE-method) was also used in the intervention [10,11] because some of the participants had experienced traumatic events. SE is a psychotherapeutic method with a psycho-physiological basis that helps to resolve and heal the symptoms of trauma. The method can be effective in reducing stress reactions for anyone who has experienced something overwhelming. It is an effective complement to Gestalt therapy.

## Method

This study is a qualitative exploration study, undertaken following psychotherapeutic intervention. The framework for the study is phenomenological-hermeneutic and inspired by the work of the French philosopher, Paul Ricoeur, about narrative and interpretation [12]. The method has been developed in Scandinavia [13,14], and used in many clinical research projects. The rationale for the choice of this method is Ricoeur’s phenomenological-hermeneutic approach where, through narrative language, meanings and significances are sought, in order to understand people and phenomena in their lived experience.

### Selection of participants

The participants were drawn from a randomised study of patients with non-specific LBP [15] who had been referred to The Spine Centre of Southern Denmark. They were 18–60 years of age and were screened for depression. Out of a total of 130 patients, 46 had mild or moderate depression, measured by the Beck Depression Inventory Scale (BDI). They were offered a series of six psychotherapeutic sessions, while simultaneously receiving cross-disciplinary somatic treatment for their LBP. All had had LBP for between 3 and 12 months and were either currently on sick leave or had been on sick leave during the previous year. Many of the patients did not complete all psychotherapeutic sessions.

In this study, participants were recruited on the basis of the following inclusion criteria: Attendance at five–six sessions; a moderate depression score of 23–30 according to the BDI (on a scale from 0–30, where 30 corresponds to severe depression); and a score for LBP of 7–10 (on a scale from 0–10, where 10 is the worst imaginable pain)*.* Out of the 46 patients, only 6 met all the inclusion criteria. The participants included four women and two men, aged between 20 and 33 years (average 29 years). All were on sick leave and had an unresolved work situation (Table 1).

**Table 1:** Overview of included patients

**Gender**	**Age**	**Depression score (BDI)**	**Pain score(0–10)**	**Therapy sessions**
Female	20	26	7	5
Female	30	25	7	6
Male	33	25	8	5
Female	31	25	9	6
Male	27	23	8	6
Female	33	28	9	5

### Data collection

The data collection is inspired by Spradley’s ethnographic method [16], which includes interaction and field notes. The data consisted of field notes in the form of written reports, drafted after each psychotherapeutic session. It was therefore the psychotherapist’s immediate perception, narrative and description of the situation, based on the patient’s narrative, statements, emotional expressions and body language. Quotations from patients were included in the written notes. The written reports from sessions on the six included patients were viewed as a continuous text that totalled 33 pages. This data became the foundation for the work and analysis in the study.

### Data processing

The processing of the data was based on the Ricoeur-inspired method that consisted of analysis and interpretation at three levels: naive reading, structural analysis and critical interpretation and discussion. The analysis and interpretation were performed by both the first and second authors.

#### Naive reading

In the first analysis, the written narratives were read and re-read several times. The process of naive reading allows for an overall grasp of the text as a whole, which gives an initial and holistic understanding of what the text contains. This is a kind of understanding where, according to Ricoeur, one sees and listens out for what moves one in the text.

#### Structural analysis

**Figure 1:**
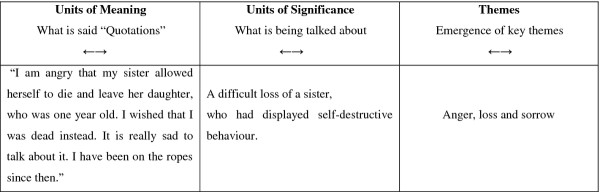
An example of the process in the structurel analyses.

Subsequently, a structural analysis was carried out, which had an explicit function. Here the text was analysed based on units of meaning, (what is said) and units of significance (what is being talked about). Topics were drawn out that were reflected in the entire text material, in order to identify some common themes. The text was analysed on the basis of not only the text in the form of direct quotations, but also the meaning behind the content, as a first interpretation of what was being talked about, in order to let themes emerge. In Figure 1, the movement from units of meaning to units of significance and to the emergence of themes is illustrated. The arrows in the diagram make it clear how there was a continuous forwards and backwards movement throughout the entire analysis process. In this way, the process is not just viewed as a progressive, linear process, but as a process where analysis and interpretation occur continuously between the parts and the whole. The process is seen as a dialectic process between explanation and understanding. Such a process helps to strengthen the rationale and arguments underpinning the themes that emerged.

### Critical interpretation and discussion

The themes were analysed, interpreted and discussed in relation to the theory and other research results.

In this final part of the analysis, there is movement from the individual patient to the study cohort, or from the specific to the general.

### Ethical considerations

As a basic starting point, the randomised study, *Individual screening-based biopsychosocial services for patients with non-specific low back pain,* was approved by the Ethics Committee of the Region of Southern Denmark (nr. VF-20060062). All participants signed a written informed consent. In the current qualitative study, no names are given. To ensure confidentiality of the participants, the study was designed according to the Ethical Guidelines for Nursing Research in the Nordic Countries (Northern Nurses´ Federation, 2003) [17]. A request to the Danish Regional Research Ethics Committee confirmed that no formal ethical approval was required for this type of study.

## Results

An overview was obtained from the naive reading. After examining the field notes in general and the participants’ quotations in particular, we found words or expressions that related to such things as sadness, failure in childhood or adolescence, abuse, anxiety, and powerlessness in the context of past experiences. These were clustered under the theme: ‘*The significance of previous experiences’.* We also found patterns related to pain, distress, insomnia, anger and irritation at not being as able as before. These were listed under the theme ‘*Restrictions in everyday life’.* Further, we discovered a number of positive statements that indicated joy, liberation, desire, strength, resolution of inner tensions, increased self esteem and desire to take action. We grouped these under the theme ’*Restoration of inner resources’*. The three themes were then analysed, interpreted and discussed. The results are reported in the following section.

### The significance of previous experiences

The analysis showed how previous experiences influenced the patients’ present life and functioning, and hence, their CLBP. For example, the loss of a close relative had triggered anger and sorrow that could have led to self-destructive behaviour, as evidenced by the following statement:

"“I am angry that my sister allowed herself to die and leave her daughter, who was a year old. I wished that I was dead instead. It is really sad to talk about it. I have been on the ropes since then.”"

It is well known that bereaved people can have an unconscious urge to ‘be in the grave themselves’ and can lose a grip on their own lives.

And another statement:

"“I took so much Somadril, just the thought of it makes me nauseous. I was saturated in medicine. I don’t understand how it’s so easy to get prescribed medicine. It was the Somadril that put me completely down in the depths. I felt that I had no idea what I was doing, when I took so much medicine. And it was not to commit suicide that I took so much and was hospitalised.”"

Throughout the quotation, a feeling of sorrow was expressed, which is thought to have caused self-destructive behaviour in the form of medicine overdose. Other themes that came up in the study were self-blame and shame. Self-blame means *I have done something wrong* and shame means *I am wrong.* Self-blame thus focuses on the action and can be healthy, if one really has done something inappropriate to another person, as this can sometimes be rectified. In contrast, however, shame is ranked with sin and inferiority [18]. From the above quotation, it seems that the patient felt it was shameful to take so much medicine, and went on the defensive by projecting the blame onto the general practitioner who prescribed the medicine, as it was too uncomfortable for the patient to admit responsibility.

Other previous experiences turned out to relate to being let down during childhood. One patient, who as an eight year old had been forced to act as an adult at home for an alcoholic mother and an absent father, made the following statement:

"“I grew up with a mother who was always drunk and from the age of eight I had to be ‘mother’ for my two younger siblings, and made sure that they did not realise that she came home drunk or saw that I cleaned up vomit in the stairway.”"

The quotation suggests a feeling of being let down. An unhealthy and reversed mother-child/father-child relationship, where the child takes responsibility for the parent, in this case the mother, is seldom something the person is conscious of, since they don’t know of any other way. From additional notes taken during the interview, it was seen how the patient, now as an adult, despite pain, depression, stress and a poor financial situation, still had to spend most of her free time being a mother for her own mother without herself receiving any support. Theoretically speaking, we can speak of the concepts of *victim, saviour* and *the violation triangle*, also called *the drama triangle*[19]. In the classic model, the child is the victim, while the mother is the saviour and the father is the violator, e.g. verbal violator. The child learns this behaviour. In the case under study, the mother was both victim and violator, and the patient saviour of her mother. In the individual therapy, the patient developed insight and showed that she was able to alter her behaviour towards her mother, and begin to take responsibility for herself and express her own needs, instead of being violated and manipulated.

The data also included a description of a sudden event that seemed to be the cause of fear of becoming paralysed. The patient fell down backwards three metres. The written quotation was as follows:

"“I fell off a front loader truck, and fell three metres. I was hoisted up and apparently was told that I was about to be lowered, but I didn’t hear that and we didn’t have eye contact, as we normally do. I didn’t register the message and suddenly I lost my balance and fell backwards. Something happened in my back and I couldn’t get up and run away. That’s what I have done all the other times I’ve fallen off ladders and the like. I wanted to crawl away, but I couldn’t move my muscles, I could only lift one leg a bit.”"

In the quotation, the patient describes how, after a sudden fall, he felt unable to move. He was terrified and went into a state of shock with the fear of becoming paralysed, although he was not paralysed. It can be assumed that he had a survival response, where the body instinctively reacted with a freeze response [20], an effect of the autonomic nervous system, where the fight or flight response was not possible. Often in such a situation, the body will reserve activated energy, that is, the energy that in a split second was activated for survival but was not used. After a violent event, there is a need for peace and calm in the following hours to regulate the autonomic nervous system. Conversely, in certain situations, symptoms can subsequently arise such as feelings of unrest, aggression, powerlessness, depression, sleep disturbance, tiredness, pain and muscle tension and a lack of capacity to carry out tasks. Ten months after the fall, in the session with the psychotherapist, following was reported: 

"“I was hospitalised, but was told that there was nothing wrong with me. I was given morphine, so finally I could sit up and the following day I was expected to go and get my own food, if I wanted something to eat."

"It wasn’t a pleasant experience, and I can’t stand hospitals - they smell of death. I have witnessed so many deaths: my friends who drove themselves to their deaths, my grandparents and a close friend who died at 19."

"So I wanted to get home quickly from the hospital and did so, but was in a lot of pain at home. At home, I cry a lot. Before the accident I was a lively guy who went round helping everyone.”"

The quotation expressed how the patient experienced a lack of calm, care and security. Furthermore, he felt that “the hospital smelled of death”, which gave him stress, anxiety and discomfort, which was why he chose to discharge himself. The fall caused a fractured vertebra and concomitant severe pain that prevented the patient from doing his usual activities; the experience of shock probably remained in the body and he subsequently developed depression and experienced powerlessness.

From a psychological viewpoint, the SE treatment was directed to help the patient to acknowledge that he had survived and to bodily integrate the fact that the danger had passed. [21]. Furthermore, it created an opportunity for him to complete his flight response which subsequently allowed the stress and fear to subside.

#### Restrictions in everyday life

The study showed how restrictions in everyday life can affect self-worth and identity. In the notes, it is stated how patients who, despite a dysfunctional family and poor social conditions, had managed to pursue an education, get a permanent job and develop a good financial situation for herself and her children. However, a workplace accident completely changed her quality of life as demonstrated in the following quotation:

"“I fell onto a wet floor at my workplace a year ago and got back pain. I get so much pain and am wiped out after doing very little. And I take strong medicine, but I can’t even sleep at night. I can’t cope with it any longer.”"

The statement shows how this patient with severe pain had sleep disturbance and felt herself to be in a powerless situation. It seems that the patient had gone into a vicious pain cycle, where pain led to insomnia and even the most minor stressor gave increased stress/high arousal causing lack of clarity and inability to cope. The patient´s level of stress was reduced via SE.

Another patient had difficulty accepting that he couldn’t do what he previously took for granted, and it seemed to affect his perception of his own identity. This is shown in the following quotation:

"“I can no longer work 100% as a craftsman. My body, my arms, where all my strength is, are now restricted because of my back.”"

With reduced functioning as expressed here in connection with back pain, a person can be affected in relation to his/her own identity, be it in relation to the family as well as to work. Self-worth can decline along with physical and psychological limitations.

And another statement:

"“I cannot feel any emotion, there is nothing that makes me sad, I used to be hot-tempered, but of course one doesn’t lose one’s temper. I don’t get it (the anger) out of my body as I used to, when I worked and played football.”"

The statement can be interpreted as conveying a repressed feeling of anger. It seems that the anger had accumulated and become either forbidden or difficult to express. Anger often creates tension in the body and exacerbates the pain. The patient worked therapeutically and phenomenologically with the pain and anger and found that the tensions in the back disappeared in the process. Another theory could be that anger can arise after an unresolved fight response [21]. The patient had on two occasions been faced with uncomfortable events, including the sudden death of close relatives. It could be suggested that the accumulation of energy was locked in the body and could have given rise to muscle tension and stress. And, as the patient described, he could not release this anger as he was unable to be physically active.

In the field notes, there were descriptions of how the patients with CLBP sometimes came with anxious, stressed and unfocused eyes but by the end of the psychotherapeutic session they were calm, and their eyes were seen to have become relaxed and clear. They became present, felt better about themselves and were more able to grasp the situation and take action.

Pain and depression can lead to feelings of inadequacy, both physically and mentally. Lack of sleep, confusion in everyday life and an uncertain future were characteristic of the participants, who described themselves as having myriad thoughts and being on the brink of not being able to cope. It is known that stress-activating life events [22], here exemplified by a complex set of symptoms related to back pain, affect depressive states.

It can be assumed that when stressors and worries become overwhelming and all internal resources have been used up, stress emerges. Stress is defined in the Encyclopaedia [23]as ‘the physiological reaction that occurs in animals and people due to threatened or actual damage to the organism or physical and/or psychological activity at the limits of the individual’s coping capacity; stress-inducing factors are collectively called stressors’. It is well known that in cases of long-term stress, people find it difficult to come out of the situation on their own [24]. If CLBP is seen as a constantly present stressor that extends into depressive symptoms, then it seems that everything becomes self-perpetuating.

#### Restoration of inner resources

The study showed, particularly in descriptions drawn from the end of the course of therapy, how an ability to tap into inner resources began to re-emerge. One female patient, who had experienced loss and sorrow and who had been self-destructive, was quoted as saying:

"“My goal is to get back to work in the town and stay living in the little community, so my children can continue to go to school there. I can feel that I’m about to wake up, instead of being the little pathetic one.”"

The study showed how, in the course of the therapy, the patient had arrived at a place where she could take responsibility and wanted to get back to work and out of the victim role. From being in a self-destructive and depressive condition, it seemed that the patient’s inner resources had now come up to the surface again and could be used.

In the field notes, another patient described how she was busy trying to satisfy everyone but herself, then at the end said:

"”I want to open my heart to myself”"

From this quotation, it can be interpreted that the patient was beginning to take care of herself. It appears that the patient had arrived at an insight where she felt that she was worth something and was ready to take herself seriously in order to move on and cope with her LBP. It was not enough that, as before, she was available to everyone else. She expressed that she experienced an inner happiness and freedom; probably a way out of her depression and pain.

For the six patients included in this study, Gestalt therapy and SE appeared to be effective for depression and therefore presumably also for pain. As the study was qualitative, it is not generalisable to people suffering from similar conditions. However, the findings are of sufficient interest to be considered in the design of future research.

## Discussion

This study shows how pain and negative experiences are repeated in different guises. It pointed to the fact that during previous experiences, the conditions that appeared to hinder the patients’ capacity to cope with life with back pain and moderate depression were loss and sorrow that caused self-destructive behaviour, including overdosing on medicine. In addition, different forms of being let down were seen, including violations and the re-experiencing of failure in a pain situation, which caused reduced self-worth, and feelings of self-blame and shame. This is supported by the literature [25] which describes how pain increases negative affect which in turn promotes association with previous unpleasant experiences. Furthermore, certain reactions to traumatic events were seen, such as anger, fear, stress, anxiety, discomfort and powerlessness. Anxiety causes certain autonomic reactions [26], which are assumed to increase the awareness of, and sensitivity to, pain and may cause increased heart rate and muscle tension. SE-method proved useful for reducing these symptoms in the included patients. Restrictions in everyday life caused distress, powerlessness, anger and the feeling of “not being able to do as before” which influenced personal identity in both family life and work life. However, inner resources were seen to re-emerge in descriptions towards the end of the course of therapy. The patients achieved insights into their own situation, began to acknowledge their own internal resources and expressed a wish to manage and look after themselves.

The results showed that CLBP and other concomitant stressors could result in tense, stressed and unfocused eyes, and how by the end of the session the eyes had relaxed and become focused and the patient had become present. Findings of stressed eyes in connection with severe stressors, pain and depression are considered to be interesting in the light of the fact that many of the patients’ reactions must be taken to be stress symptoms, e.g. anger, fear, anxiety, discomfort and powerlessness. Powerlessness, particularly in high arousal/high stress, is linked to a feeling of not being able to cope any longer. Anxiety and depression are often seen in patients with chronic pain and together they constitute a complex clinical problem [22] Pain in itself is similarly stress-inducing, since it can cause the release of increased cortisol, which again can lead to depression [27]. In addition, the patients’ frustrations regarding reduced functioning capacity and concerns about an uncertain future in terms of jobs and finances were evident. When all these stressors were at play, stress symptoms were seen.

The literature describes how depression is associated with higher pain intensity, a lower pain threshold and pain tolerance. There is some evidence that depression occurs after the development of chronic pain, and not vice versa [28]. When pain is not reduced following treatment, depression can develop. The study showed that it may be beneficial to treat depressive symptoms in parallel with other interdisciplinary treatment.

Descriptions were found in the literature [2,3] of how there is a link between LBP and psychological strain. Depression and anxiety are thought to be the most common psychological conditions associated with LBP, although depression is often seen to be atypical in the form of a so-called masked depression, where there is no feeling of dejection. The issue of interest in the current study in relation to masked depression and LBP is that a stress occurrence is more likely than an atypical depression, or it could be both. This is an aspect that the study has highlighted since the main findings are assumed to be stress symptoms.

The connection between pain, depression and stress is well known and well described [3,7,27]. It is also well known that stress in connection with for example, the death of close relatives, illness, unemployment and the like can in a broad sense cause depression in the same way as long-term severe strain. All these parameters were in play in the participants in this study, so there seemed to be justification for psychological intervention.

This study has shown how Gestalt therapy and SE were useful in contributing to the patients’ capacity to cope with CLBP. This was manifest in the patients’ renewed interest in taking responsibility for themselves, in the experience of happiness, in the feeling of being worth something, in experiencing release from muscle tension, in the reduction of stress and in the experience of being in contact with oneself and the environment.

It was not possible to find similar studies in the literature, where Gestalt therapy was used in connection with CLBP and depression. However, in the book ”Gestalt therapy på svenska” [29] the author describes how children who are let down during their childhood often present as adults to the country’s pain clinics. The effect of Gestalt therapy was reported in a meta-analysis [6] to be an effective psychotherapeutic form of therapy equal to other comparable methods within psychotherapy. SE has not yet been seen connected with CLBP and depression but is considered to be an emerging method of treatment.

The chosen method in the current study with field notes and use of narrative language was seen to be useful for those patients with CLBP and depression in articulating issues. The Ricoeur-inspired method used to analyse the data helped to give an overview, to structure, analyse and interpret the many findings that the study revealed. The strength of the research process was having an external researcher who performed the analysis and interpretation in parallel.

## Conclusions

During psychotherapeutic treatment of patients with chronic non-specific low back pain and moderate depression, diverse psychological stressors were identified, relating to both the past and the present. This study found that when pain, stress and depression become overwhelming and there are few resources available, *stress* seems to become prominent. Stressful situations can lower a person’s ability to cope with back pain.

Back pain is a stress factor in itself and, coupled with depression, these must be seen as two symptom complexes that mutually affect each other in negative ways. With the help of Gestalt therapy and the Somatic Experiencing® method, the six patients in the current study developed a greater sense of being at peace and in harmony with themselves and their environment. It seems that they achieved insight and again became capable of acknowledging and using their own internal resources. Stress responses were reduced and the patients’ capacity to take responsibility for themselves was increased.

### Perspective

As a result of the above-mentioned findings from the current study, a wider perspective could be introduced in the screening of patients with back pain for stress symptoms. Stress can be related to pain and a high depression score, and furthermore to traumatic events.

In the Spine Centre of Southern Denmark, analyzes have shown, via the screening questionnaire, Harvard Trauma Questionnaire (HTQ) Part 4, that out of 3,500 patients, 5% scored symptoms equivalent to Post Traumatic Stress Disorder (PTSD). Another 5% scored stress equivalent to sub-clinical PTSD, that is, lacking 1–2 symptoms on the full score on HTQ. This scoring was related to an event, e.g. a traffic accident, fall trauma, work injury or violence of various kinds.

More sophisticated tools for screening for stress and depression symptoms may help to lead to sub-grouping of patients and subsequent individually adapted psychological interventions. Thus, the road can be paved to increase the future success rate of treatment for patients with CLBP, an initiative that may be significant for the individual patient, the health care system and the society as a whole.

## Competing interests

The authors declare that they have no competing interests.

## Authors' contributions

Study design: HE and BDP. Data collection: HE. Analyses and manuscript preparation: HE and BDP. Both authors have read and approved the final manuscript.

## Pre-publication history

The pre-publication history for this paper can be accessed here:

http://www.biomedcentral.com/1471-2474/13/166/prepub

## References

[B1] Johansen B Mainz J Sabroe S Manniche C Leboeuf Yde C Quality Improvement in an outpatient department for subacute low back pain patients: Prospective surveillance by outcome and performance measures in a health technology assessment perspective Spine 2004 29 925 931 10.1097/00007632-200404150-00021 15082998

[B2] Joukamaa M Depression and back pain Acta Psychiatr Scand 1994 377 83 86 10.1111/j.1600-0447.1994.tb05808.x 8053373

[B3] Gatchel RJ Peng YB Peters ML Fuchs PN Turk DC The Biopsychosocial Approach to Chronic Pain: Scientific Advances and Future Directions Psychol Bull 2007 133 4 581 624 17592957 10.1037/0033-2909.133.4.581

[B4] Imes SA Clance PR Gaillis AT Atkeson E Mind´s Response to the Body´s Betrayal: Gestalt/Existential Therapy for clients with Chronic or Life-threatening Illnesses. JCLP/In Session Psychoter Pract 2002 58 11 1361 1373 Wiley Periodicals, Inc 10.1002/jclp.10084 12412147

[B5] Hoffman BM Papas RK Chatkoff DK Kerns RD Meta- Analyses of Psychological Interventions for Chronic Low Back Pain Health Psychol 2007 26 1 1 9 17209691 10.1037/0278-6133.26.1.1

[B6] Bretz HJ Heekerens HP Schmitz B A meta-analysis and the effectiveness of Gestalt therapy Z Klin Psychol Psychopathol Psychother 1994 42 3 241 260 7941644

[B7] Melzack R Pain and the Neuromatrix in the Brain J Dent Educ 2001 65 12 1378 1382 11780656

[B8] Heim C Newport DJ Bonsall R Miller AH Nemeroff CB Altered pituitary-adrenal axis responses to provocative challenge tests in adult survivors of childhood abuse Am J Psychiatry 2001 158 4 575 581 10.1176/appi.ajp.158.4.575 11282691

[B9] Clarkson P Mackewn J Fritz Perls 1993 Sage Publications Ltd, London

[B10] Levine PA Waking the Tiger – Healing Trauma 1997 North Atlantic Books, Berkeley, CA

[B11] Levine PA In an unspoken voice – How the Body Releases Trauma and Restores Goodness 2010 North Atlantic Books, Berkely, CA

[B12] Ricoeur P Interpretation Theory: Discourse and the Surplus of Meaning 1976 Texas Christian University Press, Texas

[B13] Pedersen BD Sygeplejepraksis. Sprog & erkendelse (Nursing practice. Language and cognition). PhD thesis 1999 3 University of Aarhus, Denmark

[B14] Lindseth A Norberg A A phenomenological hermeneutical method for researching lived experience Scand J Caring Sci 2004 18 145 153 Nordic College of Caring Sciences, ISSN 0283-9318 (print), ISSN 1471 6712 (online), Blackwell Publishing, Oxford 10.1111/j.1471-6712.2004.00258.x 15147477

[B15] Johansen B and Wedderkopp N Comparison between data obtained through real-time data capture by SMS and a retrospective telephone interview Chiropractic & Osteopathy 2010 18 10 10.1186/1746-1340-18-10 20500900 PMC2883994

[B16] Spradley JP Participant Observation 1980 Harcourt College Publishers, Fort Worth

[B17] Ethical guidelines for nursing research in the Nordic Countries [http://www.sykepleien.no/ikbViewer/Content/337889/SSNs%20etiske%20retningslinjer.pdf]

[B18] Wheeler G Selv og skam – en gestaltfremgangsmåde: Her and Nu. Klassiske og nye gestaltterapeutiske tekster. (Self and shame – a gestalt approach: Here and now. Classic and new Gestalt-based therapeutic texts) Redigeret of Jørgen Lumbye 2004 Paludans forlag, 3100 Hornbæk 162 186

[B19] Lynne F The Three faces of Victim. An Overviev of the Drama Trianglehttp://www.lynneforrest.com/html/the_faces_of_victim.html

[B20] Heller DP Crash Course: A self-Healing guide to Auto Accident. Trauma & Recovery 2001 North Atlantic Books, Berkeley, California

[B21] Van der Kolk BA Clinical Implications of Neuroscience Research in PTSD Ann NY Acad Sci 2006 1071 277 293 10.1196/annals.1364.022 16891578

[B22] Bech P Olsen LR Gormsen L Smerter, Angst og depression: Smerter. Baggrund, evidens og behandling. (Pain, anxiety and depression. Pain. Background, Evidence and Treatment) 2009 Redigeret af Jensen T S, Dahl J B, Arendt-Nielsen L og FADL´s Forlag A/S, København 285 298

[B23] Den Store Danske. (The Big Danish) Gyldendals åbne encyklopædi http://www.denstoredanske.dk/krop,_psyke_og_sundhed/Sundhedsvidenskab/Fysiologi/stress?highlight=stress

[B24] Prætorius NU Stress – Det moderne traume. (Stress - the modern trauma.) 2007 Dansk Psykologisk, Forlag 34 43

[B25] Eich E Rachman S Lopatka C Affect, Pain and Autobiographical Memory In J Abn Psychol 1990 99 174 178 10.1037//0021-843x.99.2.174 2348011

[B26] Jones A Zachariae B Psykologiske processers betydning for smerteoplevelsen: Smerter. Baggrund, evidens og behandling. (Psychological Processes Affect the Pain Experience. Pain. Background, Evidence and Treatment) 2009 Redigeret af Jensen T S, Dahl J B, Arendt-Nielsen L og FADL´s Forlag A/S, København 123 134

[B27] Videbech P Depression, stress and hjernefunktion: Moderne depressionsopfattelse. (Depression, stress and brain function: Modern perceptions of depression) Månedsskrift for Praktisk Laegegerning 2005 83 11 1321 1331 København23038805

[B28] Fishbain D Curtler R Rosomoff HL Rosomoff PS Chronic Pain associated depression: Antecedent or consequence of chronic pain. A review. Clin J Pain 1997 13 2 116 137 10.1097/00002508-199706000-00006 9186019

[B29] Brageé B Smarta rimmar på hjarta: Gestalt therapy på Svenska. (Pain rhymes with heart. Gestalt Therapy on Swedish) 1995 Redigeret of Inger Mannerstråle. Gestalt-Akademin in Skandinavien 194 217

